# Breast-conserving therapy versus mastectomy

**DOI:** 10.18632/oncoscience.331

**Published:** 2016-12-09

**Authors:** Marissa C. van Maaren, Philip Poortmans, Sabine Siesling

**Affiliations:** Department of Research, Netherlands Comprehensive Cancer Organisation, Utrecht, the Netherlands; Department of Health Technology & Services Research, MIRA Institute for Biomedical Technology and Technical Medicine, University of Twente, Enschede, the Netherlands

**Keywords:** observational studies, breast-conserving therapy, mastectomy, real-world population

There is an ongoing debate regarding the use of randomised controlled trials (RCTs) versus observational studies when investigating treatment effects in clinical practice [1]. This holds especially true for the comparison of breast-conserving therapy (BCT) and mastectomy (MAST), which gained much attention since the publication of our observational study in Lancet Oncology [2].

RCTs are highly appreciated as they are close to generate perfectly unbiased treatment comparison estimates. Treatment groups in a RCT are expected to be exchangeable; even when switching the treatment between the compared groups, results will be similar and are solely the effect of the treatment under study. Clinical decisions are largely based on this type of evidence. But is this always the best evidence? Is it always feasible or ethical? In the current era of personalised medicine and ‘big data’, clinical interpretation of an abundance of data (clinical reasoning) is becoming more and more crucial. It integrates all available and relevant information that may contribute to the best clinical decision-making for individual patients. This generally starts with existing guidelines, completed by evidence extracted from observational studies and clinicians' experiences [3]. Importantly, the patient's preference plays an important role in (shared) decision-making.

In general, it is difficult to translate the overall results of a RCT in the response of an individual patient to the investigated treatment. Even for patients with identical characteristics to those in the trial population, the overall treatment effect observed in RCTs would only apply if the probability of treatment benefit and detriment was equally distributed in every individual participant [3]. Often, evidence forming the basis of treatment guidelines are based on RCTs conducted a long time ago, while observational studies include a more recently diagnosed population. For BCT and MAST, the trials were all conducted in the eighties. Another important discrepancy between the RCT populations and the real-world population is the increasing share of elderly breast cancer patients in the latter. This is not only due to the ageing population, but also to early detection of breast cancer in the national screening program (which upper age limit is 75 years in the Netherlands), leading to a higher incidence in the elderly. Furthermore, diagnostic and surgical procedures as well as local and systemic therapies improved considerably. Moreover, increasing knowledge about the biological features of breast tumours led to the introduction of more advanced tumour-directed therapies. The combination of these improvements are very likely to affect survival rates. All of these matters cause results from RCTs to be of limited generalisability to the current breast cancer population. As a result, observational studies based on real-life data are increasingly conducted to provide estimates for the daily population.

This is where the battle begins. RCTs and meta-analyses of RCTs are considered to provide the highest quality of evidence, since randomisation leads to almost perfectly unbiased allocation of treatment. These types of studies thereby claim to overrule the evidence obtained by observational studies [4]. However, high-quality observational studies may provide valid estimates as well, as long as they are properly designed and interpreted with care [5]. Importantly, these kind of studies can fill the gaps left by RCTs [6].

Various RCTs confirmed that BCT has equal survival rates as MAST. These trials have been integrated in the current evidence-based guidelines that recommend either BCT or MAST in early breast cancer patients. Various observational studies concluded that BCT led to improved survival rates compared to MAST. Examples are our recently published articles regarding the effect of BCT and MAST in T1-0N0-1 and T1-2N2 breast cancer, using data of the nationwide Netherlands Cancer Registry [2, 7]. We state at least equal survival for both treatments in early breast cancer, thereby confirming results of RCTs. However, for T1N0 stage specifically, we showed that BCT led to significantly higher distant metastasis-free survival compared to MAST [2]. Of course, we discuss all potential biases and limitations in these studies and are careful with the interpretation. A large strength of these studies is the use of a nationwide population-based cancer registry, covering the entire breast cancer population. In such large populations, we are able to look at several subgroups, such as stage or age, thereby filling the gaps that are generally present in RCTs.

Both RCTs and observational studies are susceptible to particular weaknesses. RCTs contain selected patient populations not representative for the daily population, while in observational studies there is no unbiased allocation of treatment. Thus, neither of those provides flawless information. The essential question we should ask ourselves is: “What sources could have caused bias?” and subsequently, “To what extent could this bias have influenced the results, and is this clinically relevant”? The latter may be explored by sensitivity analyses for specific subgroups. Decision-making should be based on analysing possible consequences we expect to occur to the individual patient in front of us, rather than on guidelines – that are predominantly based on RCTs – only [3]. Patients should be informed as good as possible, with all relevant available information, in order to make the right decisions together with their treating physician.

Observational studies cannot replace the largely appreciated RCTs, however, RCTs should not be overrated as well, as they certainly do not make observational studies superfluous. Observational studies do not only reflect the real-life population and give information were RCTs are not possible, they can also function as hypothesis-generating research, providing questions for future RCTs [8]. Therefore, both types of studies will support clinical decision-making. The one will perfectly complement the other.

## References

[1] Chavez-MacGregor M (2016). J Clin Oncol.

[2] van Maaren MC (2016). Lancet Oncol.

[3] Sniderman AD (2013). Mayo Clin Proc.

[4] Barton S (2000). BMJ.

[5] Hershman DL (2012). J Clin Oncol.

[6] Norris S AD (2008). https://www.ncbi.nlm.nih.gov/books/NBK47093/.

[7] van Maaren MC (2016). Breast Cancer Res Treat.

[8] Song JW (2010). Plastic Reconstr Surg.

